# Self-harm and violent criminality linked with parental death during childhood

**DOI:** 10.1017/S0033291719001193

**Published:** 2020-05

**Authors:** M.J. Carr, P.L.H. Mok, S. Antonsen, C.B. Pedersen, R.T. Webb

**Affiliations:** 1 Division of Psychology & Mental Health, Centre for Mental Health and Safety, The University of Manchester and Manchester Academic Health Sciences Centre, UK; 2 National Centre for Register-based Research (NCRR), Aarhus University, Aarhus, Denmark; 3 Centre for Integrated Register-based Research (CIRRAU), Aarhus University, Aarhus, Denmark; 4 NIHR Greater Manchester Patient Safety Translational Research Centre, Manchester, UK

**Keywords:** Childhood adversity, parental death, self-harm, suicide attempt, violence

## Abstract

**Background:**

Adverse health and social outcomes are known to occur more frequently following parental death during childhood, but evidence is lacking for comparing long-term risks of internalised *v.* externalised harm.

**Methods:**

This national register-based cohort study consisted of Danish persons born 1970–2000. The Civil Registration System and National Causes of Death Register were linked to ascertain parental deaths by cause before cohort members' 15^th^ birthdays. From age 15 years, hospital-treated self-harm episodes were ascertained through linkage to the National Patient Register and the Psychiatric Central Research Register, and violent crimes were identified via linkage to the National Crime Register. Hazard ratio and cumulative incidence values were estimated.

**Results:**

Self-harm and violent criminality risks were elevated following parental death during childhood. Covariate adjustment for gender, birth year and first-degree relatives' mental illnesses attenuated these associations, although significantly heightened risks persisted. The estimated hazard ratios did not differ greatly according to which parent died, but losing both parents conferred particularly large risk increases. Risks for both adverse outcomes were higher in relation to unnatural *v.* natural parental death; violent criminality risk was especially raised among individuals exposed to parental death by unnatural causes other than suicide. The association was strongest when pre-school age children experienced parental death.

**Conclusions:**

Effective early intervention is needed to help youngsters who have experienced the death of one or both parents to develop immediate and sustained coping strategies. Enhanced cooperation between health and social services and criminal justice agencies may mitigate risks for these two destructive behaviours.

## Introduction

Around 1 in 20–30 children in Western societies experience the death of at least one parent before they themselves reach adult maturity (Melhem *et al*., 2011). It is among the most stressful life events that a person can encounter during their upbringing (Yamamoto *et al*., 1996), with a subset of affected children experiencing complicated and prolonged grief (Melhem *et al*., 2011). As well as the intrinsic distress usually caused by parental loss, these events also often confer a detrimental impact on multiple domains of a child's life by precipitating other environmental stressors. These consequent psychosocial challenges include residential moves and school transfers, reduced family income, deteriorating mental health of family members, and disruption to family dynamics through step-parenting, living with other relatives, and being adopted or fostered (Raveis *et al*., 1999; Lin *et al*., 2004).

Thus, it is unsurprising that a range of long-term adverse outcomes has been reported following exposure to parental death during childhood, including: (1) Onset of depression, anxiety disorders, post-traumatic stress disorder (PTSD), and drug & alcohol misuse (Kendler *et al*., 2002; Melhem *et al*., 2008; Brent *et al*., 2009; Otowa *et al*., 2014; Berg *et al*., 2016); (2) Suicidality (Runeson and Åsberg, 2003; Jakobsen and Christiansen, 2011; Niederkrotenthaler *et al*., 2012; Guldin *et al*., 2015; Hollingshaus and Smith, 2015; Rostilda *et al*., 2016); (3) Deficient high school grades (Berg *et al*., 2014); (4) Internalising and externalising emotional and behavioural problems (Dowdney *et al*., 1999); (5) Violent criminality (Sauvola *et al*., 2002; Wilcox *et al*., 2010); (6) All-cause and cause-specific mortality (Li *et al*., 2014).

Fatal and non-fatal suicidal behaviour following parental death has been studied extensively, with much of this evidence generated via large epidemiological studies of linked registry data in the Nordic countries. In a nested case-control study conducted using Danish national registry data, Jakobsen and Christiansen (2011) reported higher risk of attempted suicide in young people who had lost both parents compared to losing one. Risk of attempted suicide following parental suicide was reported as rising in line with increasing age at exposure in a national Swedish register-based cohort study (Niederkrotenthaler *et al*., 2012). Using national registry data pooled between the neighbouring countries of Denmark, Finland and Sweden, Guldin *et al*. (2015) reported parental death in childhood, whatever the cause, being linked with increased suicide risk in the longer term. Relative risk was higher for children whose parent died before they reached 6 years of age, and risk remained elevated for at least 25 years following the event. In another national Swedish register-based study, Rostilda *et al*. (2016) reported that experiencing the loss of one's father at pre-school age was linked with a higher risk of hospital admission for self-harm than when such a loss occurred at an older age in childhood. This applied in both male and female offspring, whereas maternal loss before school age was associated with a relatively higher risk only among affected males. The specific link between parent and offspring suicidality has been studied extensively (Runeson and Åsberg, 2003), and has been the subject of a systematic review and meta-analysis (Gueulayov *et al*., 2012).

In contrast to experiencing parental death during childhood and later suicidality risk, the association between this exposure and interpersonal violence has been reported on much less frequently in the literature. Only two previously published articles have reported on violent criminality risk linked with parental death in large epidemiological studies (Sauvola *et al*., 2002; Wilcox *et al*., 2010). In their examination of the Northern Finland 1966 Birth Cohort study, Sauvola *et al*. (2002) found a doubled risk of violent criminality, defined as including homicide, attempted homicide, assault, robbery, arson, sexual crime and violation of domestic peace, among cohort members who had experienced parental death by age 14 years. In the national Swedish register-based study conducted by Wilcox *et al*. (2010), all cohort members who experienced parental death during childhood, irrespective of cause of parental death and the specific age at which it occurred, were at elevated risk for violent criminality, with the case definition for this investigation including homicide, assault, unlawful threat, gross violation of a person's integrity, robbery, and arson. This unique study also examined suicide as an outcome, but the rarity of this external cause of death meant that it had somewhat limited power for stratifying on key modifiers, such as maternal *v.* paternal death and number of parents dying.

The national cohort study that we conducted utilised registry data from the whole Danish population to investigate whether experiencing the death of a parent during childhood was linked with elevated risks of hospital-treated self-harm and violent criminality from mid-adolescence through to early middle age. The study has added new knowledge by estimating both absolute and relative risks and by directly comparing internalised and externalised violence as correlated harmful behaviours. A sizeable proportion of individuals who harm themselves are also aggressive toward other people (O'Donnell *et al*., 2015), and this group is more likely to commit violent crimes (Sahlin *et al*., 2017). In line with previously published Scandinavian register-based studies (e.g. Jakobsen and Christiansen, 2011; Niederkrotenthaler *et al*., 2012; Li *et al*., 2014; Guldin *et al*., 2015), we assessed how the observed patterns of risk for non-fatal self-harm and violent criminality varied by: (1) Maternal *v.* paternal death; (2) Death of both parents *v.* one parent; (3) Cause of parental death - i.e. natural death, suicide, other unnatural deaths; (4) Child's gender; and (5) Age when parental death during childhood occurred. We hypothesised that the greatest elevations in risk would be found in relation to parental suicide and loss of both parents during childhood, partly because these are likely to be especially distressing and destabilising bereavement experiences (Shepherd and Barraclough, 1976; Mitchell *et al*., 2007; Jakobsen and Christiansen, 2011), and also due to familial transmission of psychopathology and suicidality (Runeson and Åsberg, 2003; Brent and Melhem, 2008).

## Methods

### Approval to conduct the study and access the registry data

The Danish Data Protection Agency approved this study, and data access was granted by the State Serum Institute and by Statistics Denmark. In accordance with Danish law, informed consent is not required for conducting register-based studies.

### Description of the study cohort

The study was conducted using whole population data extracted from interlinked national registers in Denmark. The Civil Registration System (Pedersen *et al*., 2006), which routinely captures vital information on dates of birth, death, emigration and immigration, was utilised to delineate the study cohort of persons born in Denmark between 1^st^ January 1970 and 31^st^ December 2000 and residing in Denmark on their 15^th^ birthdays. From birth or on entry to the country as an immigrant, each Danish resident is assigned a unique personal identification number, which enables accurate linkage between multiple administrative registers. Persons born outside Denmark and individuals with one or both parents born abroad were excluded from the study cohort. Implementing these restrictions precluded a potential confounding influence introduced due to elevated risk of mental disorders among first- and second-generation immigrants, as has been reported previously (Cantor-Graae and Pedersen, 2013).

### Exposure and covariate measurements

We examined all parental deaths that occurred between cohort members' births and their 15^th^ birthdays. Dates of parental death by any cause were extracted from the Civil Registration System, with specific causes of parental death additionally ascertained via the Causes of Death Register (Helweg-Larsen, 2011). We examined whether one or both parents had died during childhood, and we assessed broad categories of maternal or paternal death by underlying cause according to International Classification of Diseases coding: 8^th^ revision (ICD-8; WHO, 1971) for 1970–1993 and 10^th^ revision (ICD-10; WHO, 1993) for 1994 and onwards. The 9^th^ ICD revision was never implemented in Denmark. The following ICD coding ranges were applied to delineate three broad categories of cause-specific parental death: (1) Suicide (ICD-8 E950-E959; ICD-10 X60-X84, Y87.0); (2) Unnatural causes other than suicide (ICD-8; E800-E999, excluding E950-E959; ICD-10 V01-Y98, excluding X60-X84, Y87.0); (3) Natural causes (all codes except for ICD-8 E800-E999; ICD-10 V01-Y98). Information was extracted from the Psychiatric Central Research Register (Mors *et al*., 2011) to enable adjustment for history of secondary care-treated parental mental illness, as risks of self-harm and violent criminality linked with this exposure have been reported (Mok *et al*., 2016).

### Outcome classifications

We examined self-harm and violent criminality from mid-adolescence to early middle age. Information was extracted on hospital-treated self-harm episodes from the National Patient Register (Lynge *et al*., 2011) and from the Psychiatric Central Research Register (Mors *et al*., 2011), by applying a previously derived Danish coding algorithm (Nordentoft *et al*., 2011). To ensure consistency with our parallel examination of violent criminality, we restricted the self-harm outcome variable to the first episode occurring on or after cohort members' 15^th^ birthdays. Information regarding crimes committed from 15^th^ birthday, the age of criminal responsibility in Denmark, was extracted from the National Crime Register (Jensen *et al*., 2006). Violent crimes included homicide or attempted homicide, assault, robbery, aggravated arson, aggravated burglary, possessing a weapon in a public place, violent threats, abduction, kidnapping, terrorism, and interpersonal sexual offences. Primarily, we applied the date when the criminal act was first reported, in either the police report or at preliminary charging. If this date was unregistered, we applied the conviction date instead.

### Study design and statistical analysis

All analyses were performed using Stata v15 (Statacorp, 2017). Cohort members were followed up from their 15^th^ birthdays until the first occurrence of the adverse outcome of interest. Follow-up ended on the first of the following dates: outcome event, emigration, death, or the study's final observation date (31^st^ December 2015). We conducted time-to-event analyses to examine self-harm and violent criminality risks from mid-adolescence through to early adulthood. We fitted Cox proportional hazards models to generate hazard ratios for adverse outcomes linked with specific parental death exposure categories. At a specific point in time, an individual's ‘hazard’ is defined as the outcome rate among individuals who had not previously experienced the outcome. The hazard ratio is then calculated as the ratio of the hazard for those who experienced the death of a parent *v.* the hazard for those who did not (the reference category): the larger the hazard ratio value, the higher the relative risk for self-harm or violent criminality. We also estimated hazard ratios stratified by age at parental death, with the reference group being all cohort members who did not experience a parental death before their 15^th^ birthdays. The proportional hazards assumption for the fitted Cox regression models was examined and tested for using Schoenfeld residuals (Grambsch and Therneau, 1994). We produced both unadjusted and adjusted hazard ratios, with adjustments made for birth year (categorised as 5-year bands), and time-dependent maternal and paternal mental illness diagnostic categories, using an existing classification scheme for defining histories of mental illness in first-degree relatives (Pedersen *et al*., 2014). We did not adjust for cohort members' own psychiatric illnesses, including alcohol or drug misuse disorders, as these phenomena would lie directly on the causal pathway in many instances (Rothman *et al*., 2008).

## Results

### Descriptive statistics

The study cohort consisted of 1 698 821 persons; 871 125 males (51.3%) and 827 696 females (48.7%). Cohort members were followed from their 15^th^ birthdays over 26 273 056 person-years in aggregate. Most cohort members, 96.9% (*n* = 1 646 232), had both parents still alive at their 15^th^ birthdays; detailed prevalence estimates per exposure category are shown in Table 1. A total of 45 865 cohort members harmed themselves during follow-up, and 52 691 were convicted of committing at least one violent crime. The date when the criminal act occurred was unregistered for just 183 (0.3%) of all the violent crimes ascertained in the study. In these cases, we applied the conviction date instead.

**Table 1.** Prevalence of exposure to parental death before cohort members' 15^th^ birthdays in the national study cohort (*N* = 1 698 821)

Exposure status at 15^th^ birthday	*n*	Prevalence (%)
Any parental death	52 589	3.1
Maternal *v*. paternal death:		
Both parents alive	1 646 232	96.9
Mother died by any cause^a^	15 413	0.9
Father died by any cause^b^	36 373	2.1
Both parents died by any cause	803	0.05
Both parents died by suicide	5	<0.001
Cause of maternal death:		
Mother alive	1 682 605	99.0
Natural death	12 482	0.7
Suicide	1674	0.1
Any other unnatural cause	2060	0.1
Cause of paternal death:		
Father alive	1 661 645	97.8
Natural death	24 179	1.4
Suicide	5982	0.4
Any other unnatural cause	7015	0.4

a Mother died during cohort member's childhood, but father still alive at their 15^th^ birthday.

b Father died during cohort member's childhood, but mother still alive at their 15^th^ birthday.

### Hazard ratios (relative risks)

The upper section of Table 2 presents relative risk estimates for later self-harm. We found elevated risk across all categories of cohort members who experienced parental death during childhood. Additional covariate adjustment attenuated the estimates somewhat, although significant independent risk elevations persisted for each exposure category examined. Heightened risk was of a comparable magnitude whether a child experienced maternal or paternal death, and particularly marked elevations in risk were observed among cohort members who lost both of their parents during their childhood. As only 5 individuals had lost both parents by suicide we could not estimate relative risk for this exposure category to a reasonable degree of statistical precision. The associations observed were significantly stronger for unnatural *v.* natural parental death, whether it was the child's mother (*z* = 8.1, *p* < 0.001) or father (*z* = 9.1, *p* < 0.001) who had died. However, there was no statistically significant evidence for a difference in risk linked with parental suicide *v.* that associated with any unnatural cause of death other than suicide to mothers (*z* = −1.7, *p* = 0.09) or fathers (*z* = 0.5, *p* = 0.62). We found no evidence for effect modification by cohort members' gender in the associations observed between parental mortality during childhood and later self-harm risk, with these interaction p-values ranging from 0.13 to 0.96 across the nine exposure subgroups examined.

**Table 2.** Hazard ratios for later self-harm and violent criminality linked with exposure to parental death before cohort members' 15^th^ birthdays

Exposure status at 15^th^ birthday	*n*	Person years^a^	Rate per 1000 person years	Hazard Ratio (95% CI)	
				Unadjusted	Adjusted^b^
1. Self-harm					
*Maternal v. paternal death*					
Both parents alive	43 340	25 420 994	1.7	1.00 (*Ref*.)	1.00 (*Ref*.)
Any parental death	2525	852 063	3.0	1.75 (1.69–1.83)	1.43 (1.37–1.48)
Mother died by any cause^c^	722	249 227	2.9	1.71 (1.59–1.85)	1.44 (1.33–1.55)
Father died by any cause^d^	1739	589 685	2.9	1.75 (1.66–1.83)	1.41 (1.34–1.48)
Both parents died by any cause	64	13 151	4.9	2.90 (2.27–3.71)	1.79 (1.40–2.29)
*Cause of maternal death*					
Mother alive	45 079	26 010 678	1.7	1.00 (*Ref*.)	1.00 (*Ref*.)
Natural death	498	198 035	2.5	1.46 (1.33–1.59)	1.33 (1.22–1.45)
Suicide	122	31 277	3.9	2.37 (1.98–2.83)	1.39 (1.16–1.67)
Any other unnatural cause	166	33 067	5.0	2.90 (2.49–3.38)	1.95 (1.67–2.27)
*Cause of paternal death*					
Father alive	44 062	25 670 221	1.7	1.00 (*Ref*.)	1.00 (*Ref*.)
Natural death	978	388 817	2.5	1.48 (1.39–1.57)	1.27 (1.19–1.35)
Suicide	397	102 479	3.9	2.31 (2.10–2.55)	1.62 (1.46–1.79)
Any other unnatural cause	428	111 540	3.8	2.24 (2.03–2.46)	1.65 (1.50–1.82)
2. Violent criminality					
*Maternal v. paternal death*					
Both parents alive	49 702	25 267 369	2.0	1.00 (*Ref*.)	1.00 (*Ref*.)
Any parental death	2989	841 506	3.6	1.83 (1.76–1.90)	1.52 (1.47–1.58)
Mother died by any cause^c^	822	246 472	3.3	1.72 (1.60–1.84)	1.46 (1.36–1.56)
Father died by any cause^d^	2104	581 894	3.6	1.86 (1.78–1.95)	1.55 (1.48–1.62)
Both parents died by any cause	63	13 140	4.8	2.50 (1.95–3.20)	1.62 (1.26–2.07)
*Cause of maternal death*					
Mother alive	51 806	25 849 262	2.0	1.00 (*Ref*.)	1.00 (*Ref*.)
Natural death	555	196 470	2.8	1.42 (1.30–1.54)	1.30 (1.19–1.41)
Suicide	134	30 815	4.3	2.32 (1.96–2.75)	1.41 (1.19–1.68)
Any other unnatural cause	196	32 327	6.1	3.03 (2.64–3.49)	2.15 (1.87–2.47)
*Cause of paternal death*					
Father alive	50 524	25 513 841	2.0	1.00 (*Ref*.)	1.00 (*Ref*.)
Natural death	1169	384 804	3.0	1.55 (1.46–1.64)	1.36 (1.28–1.44)
Suicide	406	101 661	4.0	2.09 (1.90–2.31)	1.48 (1.34–1.63)
Any other unnatural cause	592	108 569	5.5	2.75 (2.54–2.98)	2.18 (2.01–2.36)

a Person years are rounded to the nearest whole year.

b Estimates adjusted for: gender; birth year; time-dependent parental mental illness diagnostic category.

c Mother died during cohort member's childhood, but father still alive at their 15^th^ birthday.

d Father died during cohort member's childhood, but mother still alive at their 15^th^ birthday.

*n* = number of persons with at least one hospital-treated self-harm episode during follow-up, or number of persons committing at least one convicted violence offence during follow-up.

The lower section of Table 2 presents relative risk estimates for later violent criminality. Risks were again consistently elevated, with and without additional covariate adjustment, in relation to each exposure category examined. The largest elevations in violent criminality risk occurred if both parents had died. The hazard ratios were significantly larger for unnatural *v.* natural death, whether the child had experienced the loss of their mother (*z* = 9.3, *p* < 0.001) or their father (*z* = 10.5, *p* < 0.001). A significantly greater risk was found for violent criminality linked with any other cause of unnatural parental death *v.* suicide in mothers (*z*  = 2.4, *p* = 0.02) or in fathers (*z* = 4.2, *p* < 0.001). Relative risk estimates for violent criminality were generally greater among female than male cohort members. Thus, as shown in Table 3, gender interaction terms were statistically significant (with observed *p*-values of 0.01 or smaller) for the following exposure categories: mother only died, father only died, any cause of unnatural maternal death other than suicide, any cause of unnatural paternal death other than suicide. Particularly marked risk elevations were observed in relation to females who were exposed to unnatural parental death with a cause other than suicide (mother: HR 5.42; 95% CI 3.94–7.46; father: HR 3.88; 95% CI 3.16–4.77).

**Table 3.** Gender-specific hazard ratios for later violent criminality linked with exposure to parental death before cohort members' 15^th^ birthdays

Exposure status at 15^th^ birthday	Hazard ratio (95% CI)		Gender interaction:	
	Males	Females	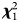	*p*-value
Maternal *v*. paternal death				
Both parents alive	1.00 (*Ref.*)	1.00 (*Ref.*)	–	–
Any parental death	1.79 (1.72–1.87)	2.29 (2.07–2.54)	18.3	<0.001
Mother died by any cause^a^	1.64 (1.52–1.76)	2.35 (1.96–2.82)	12.0	0.001
Father died by any cause^b^	1.85 (1.76–1.94)	2.24 (1.98–2.53)	7.7	0.006
Both parents died by any cause	2.37 (1.81–3.11)	3.71 (1.99–6.90)	1.5	0.22
Cause of maternal death:				
Mother alive	1.00 (*Ref.*)	1.00 (*Ref.*)	–	–
Natural death	1.37 (1.25–1.50)	1.76 (1.39–2.21)	3.6	0.057
Suicide	2.25 (1.88–2.70)	2.91 (1.83–4.62)	1.0	0.33
Any other unnatural cause	2.78 (2.38–3.26)	5.42 (3.94–7.46)	11.6	0.001
Cause of paternal death:				
Father alive	1.00 (*Ref.*)	1.00 (*Ref.*)	–	–
Natural death	1.54 (1.45–1.64)	1.73 (1.46–2.04)	1.5	0.23
Suicide	2.12 (1.91–2.35)	2.34 (1.77–3.10)	0.4	0.52
Any other unnatural cause	2.72 (2.49–2.97)	3.88 (3.16–4.77)	8.9	0.003

a Mother died during cohort member's childhood, but father still alive at their 15^th^ birthday.

b Father died during cohort member's childhood, but mother still alive at their 15^th^ birthday.

**Fig. 1.**
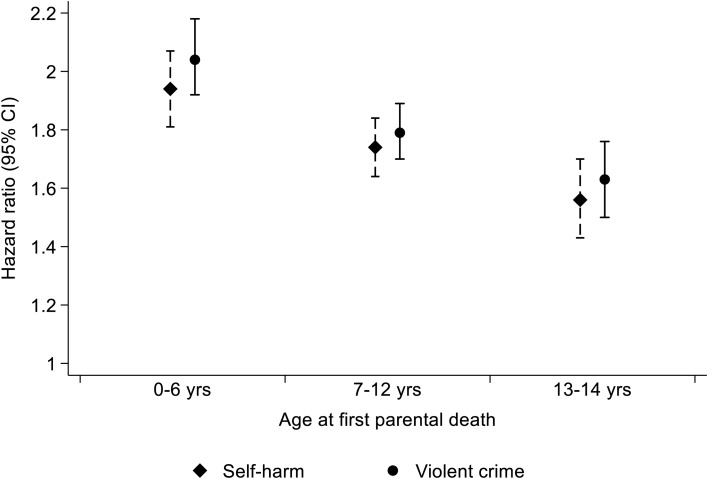
Hazard ratios (HRs) for later violent criminality and self-harm by child's age when parental death occurred.

Figure 1 illustrates hazard ratios for violent criminality and self-harm, stratified according to cohort members' childhood age at first experience of parental death by any cause. Risks for both adverse outcomes were elevated if parental death occurred at any of the three 5-year age bands between birth and 15^th^ birthday. Risk gradients were, however, clearly evident. The greatest risk elevations observed were among individuals who experienced parental death at ages 0–6 (self-harm: HR 1.94; 95% CI 1.81–2.07; violent criminality: HR 2.04; 95% CI 1.92–2.18), and the smallest were seen with parental loss at ages 13–14 (self-harm: HR 1.56; 95% CI 1.43–1.70; violent criminality: HR 1.63; 95% CI 1.50–1.76). For both adverse outcomes, there was strong evidence for a linear trend in rising risk with parental death occurring at younger age across the three age bands (self-harm, *z* = −27.8, *p* < 0.001; violent criminality, *z* = −32.5, *p* < 0.001).

### Cumulative incidence (absolute risks)

Table 4 presents gender-specific cumulative incidence percentage values for self-harm and violent criminality between cohort members' 15^th^ and 40^th^ birthdays, according to parental death during childhood exposure status. For self-harm, absolute risks were highest among males and females who lost both parents by any cause of death and those who lost their mother to an unnatural cause of death other than suicide; approximately 1 in 8 or 9 of females in these two exposure categories will have been treated in hospital after harming themselves by their 40^th^ birthdays. For violent criminality, the highest cumulative incidence values were among males who lost both parents and those who experienced the death of their mother or father by an unnatural cause other than suicide; around 1 in 5 or 6 males in these three exposure categories will have committed a violent crime on reaching age 40.

**Table 4.** Gender-specific cumulative incidence (%) of self-harm and violent criminality at age 40

Exposure status at 15^th^ birthday^a^	*n*	1. Self-harm:	2. Violent criminality:
		Cumulative incidence (95% CI)	Cumulative incidence (95% CI)
Male			
Both parents alive	844 218	3.4 (3.4–3.5)	7.1 (7.0–7.2)
One parent died by any cause	26 492	5.9 (5.5–6.2)	11.9 (11.5–12.4)
Both parents died by any cause	415	8.1 (5.2–12.0)	17.3 (13.0–22.0)
Mother died by suicide	881	7.3 (5.4–9.5)	15.5 (13.0–18.3)
Mother died by any other unnatural cause	1061	9.1 (7.1–11.4)	18.5 (15.8–21.4)
Father died by suicide	3020	7.7 (6.6–8.9)	14.1 (12.7–15.6)
Father died by any other unnatural cause	3544	7.6 (6.5–8.7)	17.6 (16.1–19.1)
Female			
Both parents alive	802 014	4.0 (3.9–4.1)	0.9 (0.9–0.9)
One parent died by any cause	25 294	6.7 (6.4–7.1)	2.0 (1.8–2.3)
Both parents died by any cause	388	12.4 (9.1–16.4)	3.0 (1.6–5.1)
Mother died by suicide	793	10.7 (8.4–13.2)	2.8 (1.7–4.3)
Mother died by any other unnatural cause	999	11.6 (9.3–14.2)	4.9 (3.4–6.8)
Father died by suicide	2962	8.9 (7.7–10.1)	2.2 (1.6–2.9)
Father died by any other unnatural cause	3471	8.3 (7.3–9.4)	3.4 (2.7–4.2)

*n* = Number of cohort members exposed.

a Exposure status categories are not mutually exclusive.

## Discussion

Self-harm and violent criminality risks between mid-adolescence and early middle age were elevated for all categories of persons who experienced parental death during childhood that we examined. Adjustment for the potential confounding influences of cohort members' gender and birth year, and also time-dependent mental illness diagnoses in mother or father attenuated the observed relative risks somewhat, although significantly heightened risk persisted nonetheless. For both adverse outcomes, the hazard ratio estimates did not differ markedly according to which parent died, but losing both parents conferred a greater risk elevation compared to experiencing the death of one parent during childhood. The associations observed in relation to both self-harm and violent criminality were stronger among young people who experienced unnatural *v.* natural death of a parent during their childhood, and there was an excess risk of violent criminality linked with any unnatural parental death other than suicide. Risks were significantly elevated at whatever age parental death occurred during childhood, but the observed associations were strongest for pre-school age children who experienced this event. For later self-harm risk, there was no evidence of effect modification by cohort members' gender for any of the exposure subgroups examined. In terms of *absolute* risk, males committed violent crimes far more frequently than females, but *relative* risk among females was greater.

This national cohort study had some key strengths shared by many epidemiological investigations conducted using interlinked Danish administrative registers, including: accurate record linkage between multiple registers; ability to account for death or emigration during follow-up; nationwide coverage; complete ascertainment of exposure and outcome variables free of information bias due to subject self-report; and abundant statistical power and precision for examining rare exposure-outcome relationships. The available registry data were, however, limited in several ways. First, the potential confounding influence of socioeconomic position could not be adjusted for in this study, because this information was unknown for approximately ninety-five percent of cohort members with a deceased parent, compared with just one percent of those with both parents alive at their 15^th^ birthdays. Second, the role of psychopathology developing during childhood could not be assessed, because mental illness diagnostic information could be ascertained solely by linkage to the Psychiatric Central Research Register, as routinely collected primary care records are unavailable to academic researchers in Denmark. Thus, only 2.8% of study cohort members had a registered mental illness diagnosis before their 15^th^ birthdays, but this information was restricted to episodes that were treated and diagnosed in secondary care psychiatric units. Third, for a very small minority of cases, the dates when violent crimes were committed were unknown. For these cases it was necessary to apply criminal conviction dates as a proxy, which resulted in crime occurrence dates being recorded at a slightly later date than when they will have actually occurred. However, this scenario applied to less than one percent of all violent crimes ascertained in the study. These rare measurement errors could therefore not materially alter the magnitude of the reported absolute and relative risk estimates.

As documented in a systematic review and meta-analysis (Geulayov *et al*., 2012), an extensive literature reports on non-fatal and fatal suicidality outcomes in people who have experienced parental suicide or attempted suicide (e.g. Runeson and Åsberg, 2003). In reviewing the literature we explored the sequelae of parental death more broadly; i.e. death of a parent by all causes and by specific causes including suicide. The findings generated by this Danish cohort study generally concurred with those from earlier studies with this broader scope, which have reported elevated risks of non-fatal self-harm (Jakobsen and Christiansen, 2011; Mittendorfer-Rutz *et al*., 2012; Rostilda *et al*., 2016), suicide (Wilcox *et al*., 2010; Guldin *et al*., 2015; Hollingshaus and Smith, 2015), and both fatal and non-fatal self-harm events examined in the same study cohort (Niederkrotenthaler *et al*., 2012).

Consistent with what we found, especially heightened risks of self-harm (Niederkrotenthaler *et al*., 2012) and of suicide (Wilcox *et al*., 2010; Guldin *et al*., 2015) have been reported in relation to decreasing age at exposure to parental death during childhood, although different age categorisations were applied in contrast with the narrow age-bands that we assessed (e.g. Wilcox *et al*., 2010, youngest exposure age group: 0–12 years). We found an excess risk of self-harm associated with the loss of both parents - a finding that has been reported previously in relation to both self-harm (Jakobsen and Christiansen, 2011) and suicide (Hollingshaus and Smith, 2015). Links between specific causes of parental death and offspring risk have been examined in earlier studies. Excess suicidality risks associated with parental suicide (Wilcox *et al*., 2010; Niederkrotenthaler *et al*., 2012; Guldin *et al*., 2015) and all unnatural / substance abuse deaths combined (Rostilda *et al*., 2016), as compared with more modest risk elevations linked with parental natural death, have also been reported previously. Thus, for example, a collaborative Nordic registry study that pooled national registry data from Denmark, Finland and Norway, reported an incidence rate ratio of 3.4 (95% CI 2.6–4.5) among persons bereaved as a child by parental suicide *v.* 1.8 (95% CI 1.5–2.1) for those who experienced any other cause of parental death whilst growing up (Guldin *et al*., 2015).

A notable finding from our study is the excess risk of violent criminality linked with any cause of unnatural parental death other than suicide. In the absence of any previously published evidence to draw upon, it seems reasonable to speculate that, in some cases, the impact on children of a parental suicide could have been mitigated to a degree by an expectation that such an event might occur, with protective measures perhaps initiated proactively by family and/or services to facilitate coping mechanisms in the affected children. On the other hand, parental deaths by any cause of unnatural death other than suicide could include a disproportionate number of unpredictable and especially harrowing events occurring accidentally, by undetermined intent or willfully by assault in families characterised by chaotic lifestyles, alcohol or drug misuse, and interpersonal violence. Such extreme scenarios might have an even more harmful impact on the developing child or adolescent than parental suicide.

Our literature review indicated that the only previously published research to have examined both internalised and externalised violence in the same study cohort was conducted using Swedish national registry data (Wilcox *et al*., 2010). Whereas we found particularly strong associations with later violent offending risk among persons who experienced the unnatural death of a mother or father at ages 0–14, the Swedish investigators reported a more homogeneous set of relative risks in relation to exposure to parental death by cause at ages 0–25 (suicide: 1.4; 95% CI 1.2–1.6; accidental death: 1.4; 95% CI 1.2–1.6; other causes of parental death: 1.5; 95% CI 1.3–1.8). Furthermore, their study did not report on gender differences, on maternal *v.* paternal death, or on whether one or both parents died during childhood. Thus, we do not know if the markedly greater relative risk estimates for violent criminality that we observed among females in Denmark were also present in a similar cohort from the neighboring country of Sweden. This earlier Nordic registry study, did, however, indicate that relative risks for violent criminality did not vary according to cohort members' age at experiencing parental death.

A systematic review of traumatic experiences in early childhood has emphasised the importance of including pre-school age as a specific exposure category when examining the sustained impact of childhood adversities on developmental trajectories (Lieberman *et al*., 2011). We therefore need to understand why stronger links with later self-harm and violent criminality risks were observed when parental death had occurred during infancy or at pre-school age. Many of these individuals are likely to have been particularly vulnerable due to their limited coping skills at such young age and an absolute dependence on their primary caregiver to shield them from harm (De Young *et al*., 2011). Furthermore, historically a sizeable proportion of clinicians may have held an opinion that the very youngest children could not develop clinically significant psychopathology. This flawed and outdated notion may have contributed to under-detection of psychological distress and emerging psychiatric illness among some of these children (De Young *et al*., 2011) – a problem compounded by a dearth of clinicians specifically trained and sufficiently experienced in assessing complex needs and effectively treating symptomatology at such a young age (Lieberman *et al*., 2011).

It is challenging to elucidate causal pathways when investigating the sustained impact of experiencing parental death during childhood. Further research is therefore needed to disentangle acute effects specific to these bereavements at young age *v.* those due to pre-existing familial psychosocial adversities or genetic transmission of psychopathology (Thapar *et al*., 2007) and related impulsivity (Bezdjian *et al*., 2011; Bevilacqua and Goldman, 2013), to thereby enhance understanding of the determinants of later self-harm and interpersonal violence. Investigations of specific biomarkers or polygenetic risk scores, as well as environment factors, may indicate how these complex mechanisms combine to influence risk at whole population level. Whilst the purpose of the study that we herein report was to discern the generalised association between parental death and risks for later adverse outcomes, it is important to acknowledge that individual children who experience parental death have distinct biological, cognitive, and psychological characteristics that will determine their capacity to cope with such a painful loss. Thus, unmeasured qualities of cohort members before they experience parental death, and their relationship with the deceased parent, will modify risks for these adverse outcomes. A better understanding is therefore needed of the factors that promote resilience in some individuals who experienced parental death during childhood, and which of these factors can potentially be ‘learned’ by less resilient individuals.

## Conclusions

Effective early intervention is indicated to help affected children and adolescents to develop coping strategies to deal with the immediate crisis and newly altered family or school environments, whilst recognising that manifestations of the harmful impact of a parental death could be delayed until some years after it occurred. Bereavement due to parental suicide is linked with elevated risks for subsequent harmful behaviours in excess of the risks associated with the natural death of a parent. However, the findings from this study emphasise that parental deaths from unnatural causes other than suicide are also more likely to engender serious negative consequences in later life, and risks of adverse outcome linked with experiencing death of a parent from these causes may be even higher than those associated with parental suicide. A decade ago, suicide bereavement was perceived as being a neglected problem (Ajdacic-Gross *et al*., 2008), but it has now moved to the forefront of the research and policy agenda (Maple *et al*., 2018). Our findings emphasise that people bereaved by deaths from unnatural causes other than suicide should not be overlooked in the development of supportive interventions for affected families. Particularly attentive monitoring and robust care and support are needed for young persons who lost both of their parents whilst they were growing up. As some young persons who experienced parental death during childhood are more likely to harm themselves or other people, enhanced levels of cooperation between health and social services and criminal justice agencies may mitigate the elevated risks for these two detrimental behaviours as these individuals transition from adolescence to adult maturity.
